# Dysregulation of Hepatitis B Virus Nucleocapsid Assembly *in vitro* by RNA-binding Small Ligands

**DOI:** 10.1016/j.jmb.2022.167557

**Published:** 2022-03-24

**Authors:** Nikesh Patel, Fardokht Abulwerdi, Farzad Fatehi, Iain W. Manfield, Stuart Le Grice, John S. Schneekloth, Reidun Twarock, Peter G. Stockley

**Affiliations:** 1 Astbury Centre for Structural Molecular Biology, University of Leeds, Leeds LS2 9JT, UK; 2 Center for Cancer Research, National Cancer Institute, Frederick, MD 21702-1201, United States; 3 Department of Mathematics, University of York, York, YO10 5DD, UK; 4 York Cross-disciplinary Centre for Systems Analysis, University of York, York, YO10 5GE, UK; 5 Department of Biology, University of York, York, YO10 5DD, UK

**Keywords:** packaging-signal mediated assembly, directly-acting anti-virals, hepatitis B virus

## Abstract

RNA sequences/motifs dispersed across the genome of Hepatitis B Virus regulate formation of nucleocapsid-like particles (NCPs) by core protein (Cp) *in vitro,* in an epsilon/polymerase-independent fashion. These multiple RNA Packaging Signals (PSs) can each form stem-loops encompassing a Cp-recognition motif, -RGAG-, in their loops. Drug-like molecules that bind the most important of these PS sites for NCP assembly regulation with nanomolar affinities, were identified by screening an immobilized ligand library with a fluorescently-labelled, RNA oligonucleotide encompassing this sequence. Sixty-six of these "hits", with affinities ranging from low nanomolar to high micromolar, were purchased as non-immobilized versions. Their affinities for PSs and effects on NCP assembly were determined *in vitro* by Surface Plasmon Resonance. High-affinity ligand binding is dependent on the presence of an -RGAG-motif within the loop of the PS, consistent with ligand cross-binding between PS sites. Simple structure−activity relationships show that it is also dependent on the presence of specific functional groups in these ligands. Some compounds are potent inhibitors of *in vitro* NCP assembly at nanomolar concentrations. Despite appropriate logP values, these ligands do not inhibit HBV replication in cell culture. However, modelling confirms the potential of using PS-binding ligands to target NCP assembly as a novel antiviral strategy. This also allows for computational exploration of potential synergic effects between antiviral ligands directed at distinct molecular targets *in vivo.* HBV PS-regulated assembly can be dysregulated by novel small molecule RNA-binding ligands opening a novel target for developing directly-acting anti-virals against this major pathogen.

## Introduction

As of 2016, theWHO estimated that over 2 billion individuals were infected with Hepatitis B Virus (HBV), which is currently the main risk factor for liver cancer. Its heavy annual death toll and resultant economic burdens arise within the cohort of ~260 million people who suffer from chronic infections,^1,2^ for which there is currently no cure. These patients typically suffer cycles of asymptomatic liver inflammation, which over decades can lead to hepatocellular carcinoma, cirrhosis and death. Despite the availability of an effective vaccine,^3^ it is not universally deployed, allowing ~1 million new infections to occur annually. Many of these cases are due to vertical transmission from mother to child, a route that frequently allows the infection to progress to the chronic state. Current clinical therapy for HBV infections targets the reverse transcriptase (RT) function of the virally-encoded polymerase (Pol).^4,5^ Treatment with nucleot(s)ide analogues is not curative, is required throughout the lifetime of the patient, and only lowers the eventual death toll via liver disease by 2–4 fold.^6^ Interferon therapy is also used as an adjuvant for the immune system but has had limited success in reducing HBV levels, and produces undesirable side effects.^4,5^ Cp-binding allosteric modulators (CPAMs) that inhibit correct assembly of the NCP are also under development,^7–9^ as are inhibitors of the RNase active site of Pol.^10,11^ The WHO has declared a Global Challenge to make HBV a fully treatable disease by 2030.^2^

HBV is a para-retrovirus (Sup Figure 1), i.e. a dsDNA virus which initially packages a positive-sense, single-stranded (ss) ~3500 nt RNA, termed the pre-genomic RNA (pgRNA), into a *T* = 4 NCP.^12–14^ NCPs with *T* = 3 architecture are also produced but whether they contribute to infection is not known. The pgRNA carries a 5' cap and a 3' poly-A tail,^15,16^ and forms a complex with a molecule of the virally-encoded Pol bound to the ε RNA stem-loop.^17–20^ The Pol enzyme reverse transcribes the pgRNA, whilst degrading it, and then copies the ssDNA strand formed, all within the confines of the NCP.^21–23^ Virion formation, budding and infection are complex processes,^24^ resulting in formation of a relaxed, circular dsDNA genome (rcDNA) that is delivered to new host cells. This rcDNA is repaired by host enzymes, generating a covalently-closed circular DNA (cccDNA),^25,26^ from which the pgRNA and sub-viral mRNAs for expression of HBV proteins are transcribed.^12,20^ Persistence of the cccDNA ensures the longevity of HBV infection.

Given the initial requirement to package a ssRNA genome, we previously explored whether this virus assembles its NCP using the RNA Packaging Signal (PS)-mediated mechanism that we identified for spherical ssRNA viruses from a number of different viral families^27–33^. These viral genomes encompass multiple, dispersed sequences, often in the form of stem-loops, that act collectively in binding CPs, thus regulating formation of the infectious virion^27–29,31,32,34,35^. The differing PS sites within a gRNA have differential affinities for their cognate CPs defining a preferred assembly pathway (a Hamiltonian path).^33^

**Figure 1:**
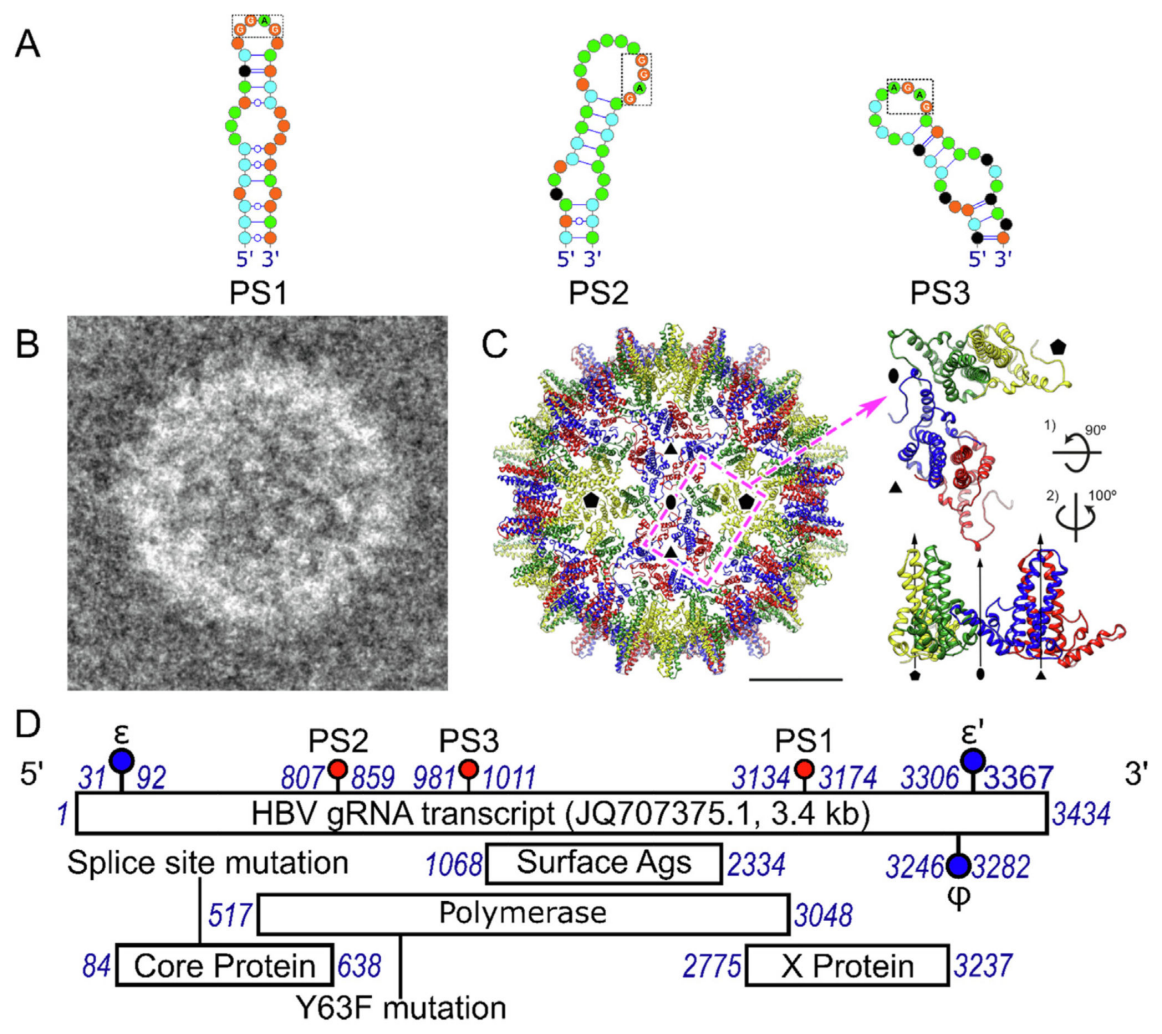
Components of the in vitro HBV NCP assembly system. (A) *Left to right:* PS1, PS2 and PS3 (NC003977.1 strain) as identified from previous SELEX studies^31^ presenting the Cp-recognition motif, −RGAG− within the apical loop. (Note nucleotides, here & throughout, are shown as coloured circles - green = A; orange = G; black = C; & cyan = U. Watson-Crick base pairs are indicated as lines, interrupted by circles for G-U pairs. (B) *Left:* negatively-stained, electron micrograph of a *T* = 4 NCP reassembled with the gRNA transcript used here. (C) *Left to right:* Front-half of the cryo-EM reconstruction of the reassembled NCP from (B) showing the N-terminal globular portions of the four quasi-equivalent monomers; A in yellow; B in green; C in blue & D in red (PDB ID: 7ABL).^36^ The dashed magenta rectangle indicates the asymmetric unit composed of 2 Cp dimers shown in orthogonal views to the *right.* (D) Genetic map of the gRNA of HBV strain JQ707375.1, showing the locations (nucleotide numbers in blue italics) of its ORFs, together with the RNA PSs (PS1, PS2 and PS3, highlighted as red lollipops), and the ε and ψ (blue lollipops) stem-loops, implicated in polymerase binding and gRNA compaction.^36^

RNA SELEX^31^ against Cp from HBV strain NC_003977.1, and bioinformatics analysis of the resulting Cp-binding aptamers, was used to identify 9 putative PSs that are conserved across the pgRNA sequences of 14 randomly selected HBV strains from GenBank.^27^ All of these sites are highly conserved in each of these genomes, consistent with their functions as PSs, and the three PSs in strain NC_003977.1 best matched by the aptamers are completely conserved (PSs1-3, Figure 1A). When presented within oligonucleotides, each triggers sequence-specific assembly of Cp dimers *in vitro* into mostly *T*= 4 NCPs. Sequence variants of homologous PS sites within genome length transcripts (gRNA) of strain JQ707375.1, which was not part of the previous analysis, also regulate efficient NCP assembly (Figure 1B−D) in the absence of the terminal modifications of the pgRNA and bound Pol.^36^ PS1 is most important for NCP assembly regulation in these *in vitro* assays. A combination of cryo-EM reconstruction and RNA X-ray footprinting of the reassembled NCPs^36,37^ confirms that the pgRNA-Cp interaction includes the PS1 Cp-recognition motif (−RGAG−), as expected for PS-mediated assembly.

Here we describe the identification of uncharged, small molecule, PS1-binding ligands, some with nanomolar affinities. Several of these are potent inhibitors of NCP formation *in vitro,* implying that its assembly regulation via gRNA-Cp interactions could be a drug target.

## Methods

### Cloning, expression and purification of HBV Cp dimer

HBV Cp was expressed from a pET28b plasmid in *E. coli* BL21(DE3) cells and purified as NCPs.^31,38^ NCPs were dissociated into Cp dimers using 1.5 M guanidinium chloride as previously described.^31,38^ The HBV Cp dimer concentration was determined spectrophotometrically (ε_280_ of Cp dimer = 55,920 L mor^−1^ cm^−1^) in a Nanodrop™ One. Fractions with an *A*_260/280_ ratio of ~0.65 or lower were used in reassembly assays. Absorbance values at 260 and 280 nm were corrected for light-scattering throughout, using the absorbance values at 310 and 340 nm, as previously described.^39^

### Preparation of genomic RNA

A wild-type HBV gRNA construct was assembled using a clone purchased from ATCC^®^ (pAM6 39630™, strain acc. no JQ707375.1). The pgRNA precursor sequence was copied in a modular fashion using PCR and cloned into the correct order within a pACYC184 vector, between the *BspHI* and *HindIII* sites using a Gibson reassembly^®^ Master Mix, according to the manufacturer's protocol (New England Biolabs). Transcription of the RNA was carried out using a Highscribe T7 High-yield RNA synthesis kit (NEB), after linearisation of the DNA plasmid using *HindIII.* RNA was annealed by heating to 70 °C for 90 sec followed by cooling slowly to 4 °C in a buffer containing 50 mM NaCl, 10 mM HEPES and 1 mM DTT at pH 7. Products were assessed using a 1% (w/v) denaturing formaldehyde agarose gel (Sup Figure 2). RNA concentration was estimated using *A*_260_ values (*ε*_260_ of gRNA = 32, 249 mM^−1^ cm^−1^).

### Small molecule microarray screening

SMM screening was performed as previously described.^40^ Briefly, Corning GAPS II slides were functionalized with an isocyanate group using an established procedure.^41^ Slides were printed using an ArrayIt NanoPrint LM60 microarrayer, and compounds were printed as 10 mM stock solutions in DMSO with two replicate spots. After printing, slides were incubated with fluorescently-labelled PS1 RNA purchased from Dharmacon in RNase-free 1 × PBS buffer (12 mM NaH_2_PO_4_, 137 mM NaCl, 3 mM KCl, pH 7.4). After washing, slides were imaged with an Innopsys 1100 scanner. Composite Z scores were generated for each compound. Hits were characterized as (1) having a Z score >3; (2) having an increase of Z-score of 3 relative to a buffer-incubated control; and (3) having a coefficient of variance (CV) of <120% for replicate spots.^40^ Finally, hits were filtered for selectivity against all other RNAs screened previously in the SMM format in the Schneekloth laboratory.

### Assaying ligand affinities for PS RNAs

#### RNA preparation

5'-Amino-labelled RNA oligonucleotides (Sup Table 1, Figure 3) were purchased from IDT and biotinylated covalently by rolling at room temperature for 4 h in 100 mM sodium borate buffer (pH 8.0), with a 100-fold molar excess of EZ-Link Sulfo-NHS-LC-LC-Biotin (Thermo Fisher). Biotinylated oligonucleotides were extracted from a 10% (v/v) denaturing acrylamide gel via a 'crush and soak' method. Gel slices corresponding to the desired oligonucleotides were excised and eluted three times into separate aliquots (100 μL) of 10 mM TE buffer over 3 h. Aliquots were combined and precipitated overnight using 50%(v/v) isopropanol, 0.3 M NaOAc and 1/100 volume RNA grade glycogen at −20 °C. Biotinylated, precipitated RNA was eluted into 100 μL ddH_2_O.

#### Determining ligand affinities using surface plasmon resonance (SPR)

Biotinylated RNA oligonucleotides were immobilised onto the surface of streptavidin-coated chips (GE Healthcare) in a BIAcore T200 at a concentration of 100 nM. Buffer flow was set at 5 μL/min over 15 min contact time, and the surface of the chip saturated with the RNA oligonucleotides, as determined by a plateau in the resulting refractive index change. Small molecular weight compounds stored in DMSO at a concentration of 10 mM, were used at initial concentrations of 75, 100, 150, 200 and 300 μM. Further measurements at lower concentrations were taken where *K_D_* measurements were lower than 75 μM, dropping as low as 1 nM for compound #63. These were stored in a chilled compartment at 9 °C within a skirted, 384-well plate (GE Healthcare) before being washed over the chip surface in a buffer containing 20 mM HEPES (pH 7.5), 250 mM NaCl, 2%(v/v) DMSO and 0.1%(v/v) Tween20, at a rate of 30 mL/min for 6 min at 37 °C, allowing for an association time of 1 min followed by dissociation over 5 min. Solvent corrections to account for variable concentrations of DMSO were performed according to manufacturer's instructions. Data were plotted with the best-fitting model as determined by the chi^2^ values. All fits used the two-state model available in the Biacore T200 evaluation software, where A is the analyte (compound), and B is the immobilised RNA. Complex formation between A and B results two kinetically distinct products, denoted as AB and AB* below (GE Healthcare).

(1) $$A+B\overset{ka1}{\underset{kd1}{⇌}}AB\overset{ka2}{\underset{kd2}{⇌}}AB*$$

A two-state model (1) was used to fit the SPR curves, so that the association (*k_a1_, k_a2_*) and dissociation (*k_d1_, k_d2_*) rate constants of complexes AB and AB* are given by:

(2) $$dR_{AB}/dt=\left(k_{a1}\cdot C_{A}\right)\cdot \left(R_{\text{max}}=R_{AB}-R_{AB*}\right)-\left(k_{d1}\cdot R_{AB}\right)-\left(k_{a2}\cdot R_{AB}\right)+\left(k_{d2}\cdot R_{AB}*\right)$$

(3) $$dR_{AB*}/dt=\left(k_{a2}\cdot R_{AB}\right)+\left(k_{d2}\cdot R_{AB}*\right)$$

Eq. (3) defines the rate of formation of each complex, AB (2) and AB* (3), directly from the SPR response curves obtained. R is the concentration of the complex (subscript) and C_A_ is the concentration of the analyte.

(4) $$K_{D}=\left(k_{a1}/k_{d1}\right).\left(1+k_{a2}/k_{d2}\right)$$

Dissociation and association constants from (2) and (3) were used to calculate an affinity constant (4).^42,43^

#### HBV NCP reassembly

178 μL of 1.1 nM heat-annealed gRNA in a buffer containing 20 mM HEPES (pH 7.5), 250 mM NaCl, and 5 mM DTT was incubated in each well of a 96-well plate at room temperature for 30 min. 2 μL of DMSO ± compound was then added, and a further equilibration was performed for 30 min. Cp dimer in dissociation buffer (as above) was then titrated into the RNA using a Biomek 4000 liquid-handling robot (Beckmann Coulter), step-wise up to a ratio of 1200:1 (10 discrete 2 μL solution aliquots, using the following Cp dimer concentrations (in brackets), were then added: 1 (100 nM), 10 (1 μM), 25 (2.5 μM), 75 (7.5 μM), 120 (12 μM), 240 (12 μM), 480 (24 μM), 720 (24 μM), 960 (24 μM) and 1200 nM (24 μM) Cp dimer). The final volume in each well was 200 μL, i.e. a cumulative volume of 19.2 mL/plate. The final concentrations of RNA and Cp were 1 nM and 1.2 μM, respectively. Cp aliquots were calculated such that they reached a maximum of 10% of the final volume, maintaining the GuHCl concentration <0.15 M. Following incubation at room temperature for 1 h, the samples were pooled and concentrated to a final volume of 2 mL. The ***A***_260/280_ ratio was measured using a Nanodrop™ One (Thermofisher Scientific), and the RNA concentrations calculated using the corrected absorbance value at 260 nm. Each sample was split into 2, with one half treated with 1 μM RNase A, and incubated overnight at 4 °C. After incubation, 5 μL of each sample was visualised by negative stain electron microscopy (nsEM) to assess particle yield, shape and "completeness". The remaining samples were analysed by application to a TSK G6000 PWXL column (Tosoh), in a buffer containing 20 mM HEPES (pH 7.5), 250 mM NaCl and 5 mM DTT, attached to a SEC-MALLS system (ÄKTA Pure (GE Healthcare) connected to an Optilab T-REX refractometer and miniDAWN^®^ multiple angle laser light-scatterer fitted with a Wyatt QELS DLS module (Wyatt Technology)). Light-scattering peaks were collected and concentrated to ~1 ml, where their RNA content, was estimated using their ***A***_260_ relative to the starting material.

#### Computational modelling of ligand inhibition of NC assembly

Assembly kinetics were modelled via a set of reactions adapted from our recent intracellular model for HBV infection,^44^ that includes the roles of the three evolutionarily conserved packaging signals (PS1-PS3) in the gRNA during virion assembly.^31^ We assume that all three PSs interact with Cp dimers and PS-targeting compounds according to the following reactions:

$$\begin{array}{l} \begin{array}{cc} \text{gRNA+X}⇌{\text{gRNA:X}}_{i} & 1\leq i\leq 3 \end{array}\\ \begin{array}{cc} \text{gRNA}:{\text{X}}_{i}+\text{X}⇌\text{gRNA}:{\text{X}}_{i}:{\text{X}}_{j} & j \end{array}\in \left\{1,2,3\right\}-\left\{i\right\}\\ \text{gRNA}:{\text{X}}_{i}:{\text{X}}_{j}+\text{X}⇌\text{gRNA}:{\text{X}}_{1}:{\text{X}}_{2}:{\text{X}}_{3}, \end{array}$$

where X denotes either a Cp dimer (C) or a compound (d), and an index refers to the PS site where binding occurs. For example, gRNA :C_1_ : C_2_ : d_3_ indicates C bound to PSs 1 and 2, and a drug molecule to PS3. The gRNA :C_1_ : C_2_ : C_3_ complex will then recruit 117 Cp dimers at a rate κ to build an intact particle via recruitment of further CP dimers:

$$\begin{array}{l} \text{gRNA}:{\text{C}}_{1}:{\text{C}}_{2}:{\text{C}}_{3}+\text{C}\to \text{gRNA}:4\text{C}\\ \begin{array}{cc} \text{gRNA}:j\text{C}+\text{C}\to \text{gRNA}:\left(j+1\right)\text{C} & 4\leq j\leq 119. \end{array} \end{array}$$

In order to model malformations caused by drug molecules, we introduce reactions in which the number of Cp dimers to be recruited is limited to *n_c_*. The latter is estimated based on the experimentally determined hydrodynamic radii (*R_h_*) of the malformed particles (Table 2). In particular in the compound-free case we have *R_h_* = 18.2nm and each nucleocapsid contains 120 Cp dimers. As the surface of a sphere is equal to 4*πr*^2^, we assume that the number of Cp dimers is in a good approximation proportional to $R_{h}^{2}$. Therefore, we use $n_{c}=120\times {\left(\frac{R_{h}}{18.2}\right)}^{2}$ for the average number of Cp dimers in a malformed particle. For example, *n_c_* ≈ 114 for *R_h_* = 17.7 nm. A Gillespie algorithm was used to perform stochastic simulations of the system, resulting in the time-course of virus build up in the absence and presence of the drug molecules.

#### Parameter value estimation

178 μl of 1.1 nM gRNA in the drug-free scenario corresponds to 1.179 × 10^11^ copies of gRNA. The simulation mimics the experimental protocol for Cp dimer titration with step-wise addition in 10 discrete steps with 10 minutes intervals up to a ratio of 1200:1. The numbers of Cp dimers added at consecutive steps were; 1.2 × 10^11^,9× 1.2 × 10^11^,15 × 1.2 × 10^11^,50× 1.2 × 10^11^,45× 1.2 × 10^11^,120 × 1.2× 10^11^,240 × 1.2 × 10^11^ 240 × 1.2 × 10^11^,240× 1.2 × 10^11^, and 240× 1.2 × 10^11^. After the equivalent of one hour incubation time, assembly reactions are started with recruitment rate κ equal to 10^6^ M^−1^ s^−1^^45^ and PS binding affinity for Cp of 4 nM.^44^ As 1 *A*_260_ Unit = 40 μg/ml, and assuming a volume of 100 μl and an average weight of a nucleocapsid of 4 MDa (6.6422 x 10^–18^ g),^46^ we obtain $n_{p}=0.04\times OD\times \frac{1}{6.6422\times 10^{-18}}\times 10^{-4}=6\times 10^{11}\times OD$ for conversion of OD values to the number of assembled particles (*n_p_*). Since after nuclease treatment (160 minutes post the experiment start) *A*_260_ is 0.12, the number of fully formed nucleocapsids is equal to 7.2 × 10^10^. We fitted the forward rate of Cp dimers for binding to PSs (*f_nuc_*) to match this value, giving us 336 M^−1^ s^−1^.

In the presence of 1 nM of Compound #63, *A*_260_ is 0.028 after nuclease treatment. Using the formula above, *n_p_* ≈ 1.7 x 10^10^. We explored different forward rates for the binding of Compound #63 to PSs (*k_f_*). For *k_f_* < 5 μM s^−1^, the number of fully formed particles is > 1.7 x 10^10^. Increasing *k_f_* from 5 to 10 μM^−1^ s^−1^ reduces the number of nucleocapsids to 1.4 x 10^10^. However, further increasing *k_f_* does not reduce the number of nucleocapsids. We therefore show results for *k_f_* = 10 μM^−1^ s^−1^, and use this value for other compounds, which is consistent with an estimate of 1 – 10 μM^−1^ s^−1^ for other molecules.^47^ This model, combined with a post HBV-infection intercellular model,^48^ allows the impacts of various antiviral ligands to be estimated without recourse to animal experiments. In particular, it allows us to look at the effects of synergy between DAAs.

#### Fluorescence anisotropy NCP assembly assay

178 μL of 1.1 nM heat-annealed gRNA or 16.5 nM heat-annealed PS1(NC_003977.1) in a buffer of 20 mM HEPES (pH 7.5), 250 mM NaCl and 5 mM DTT was incubated in wells of a 96 well plate at room temperature for 30 min. Each nucleic acid was fluorescently labelled at the 5' end using an AlexaFluor SDP ester as previously described.^31^ 2 μL of DMSO ± compound was added, and a further equilibration was performed for 30 min. Cp dimer in dissociation buffer (as above) was then titrated into the RNA using a Biomek 4000 liquid-handling robot (Beckmann Coulter), step-wise up to a ratio of 1200:1 (10 discrete 2 μL solution aliquots, using the following Cp dimer concentrations (in brackets), were then added: 1 (100 nM), 10 (1 μM), 25 (2.5 μM), 75 (7.5 μM), 120 (12 μM), 240 (12 μM), 480 (24 μM), 720 (24 μM), 960 (24 μM) and 1200 nM (24 μM) Cp dimer). RNA only controls were performed, with dissociation buffer added without Cp dimer. The final volume in each well was 200 μL. Fluorescence anisotropy was measured after each addition using a POLARstar OMEGA (BMG Labtech, Aylesbury). The final concentrations of RNA and Cp were 1 nM and 1.2 μM, respectively. Cp aliquots were calculated such that they reached a maximum of 10% of the final volume, which keeps the GuHCl concentration <0.15 M. Each well was then treated with 1 μM RNase A, and incubated at room temperature for 1 hour, after which a final fluorescence anisotropy reading was taken. These values were normalised with respect to the initial reading, and the anisotropy change calculated as the final RNase treated sample – the starting fluorescence anisotropy of the RNA.

## Results

### Identification & characterization of PS1 binding compounds

A 47-nt long oligonucleotide, capable of folding into a stem-loop encompassing the NC_003977.1 PS1 sequence (Figure 1A), was 5'-labelled with the red fluorescent dye (AlexaFluor 647 for SMM screening. A library of ~20,000 drug-like molecules covalently-linked onto an isocyanate-functionalized glass surface^40^ was incubated with a solution of labelled RNA. A second copy of the same slide was incubated with the buffer used to anneal the RNA. These signals were used to correct for auto-fluorescence by immobilised compounds. After two buffer washes, slides were scanned at 635 nm and PS1-binding compounds identified, as described in Methods, identifying 72 ligands that bind HBV PS1 (Sup Figure 3).

**Figure 2:**
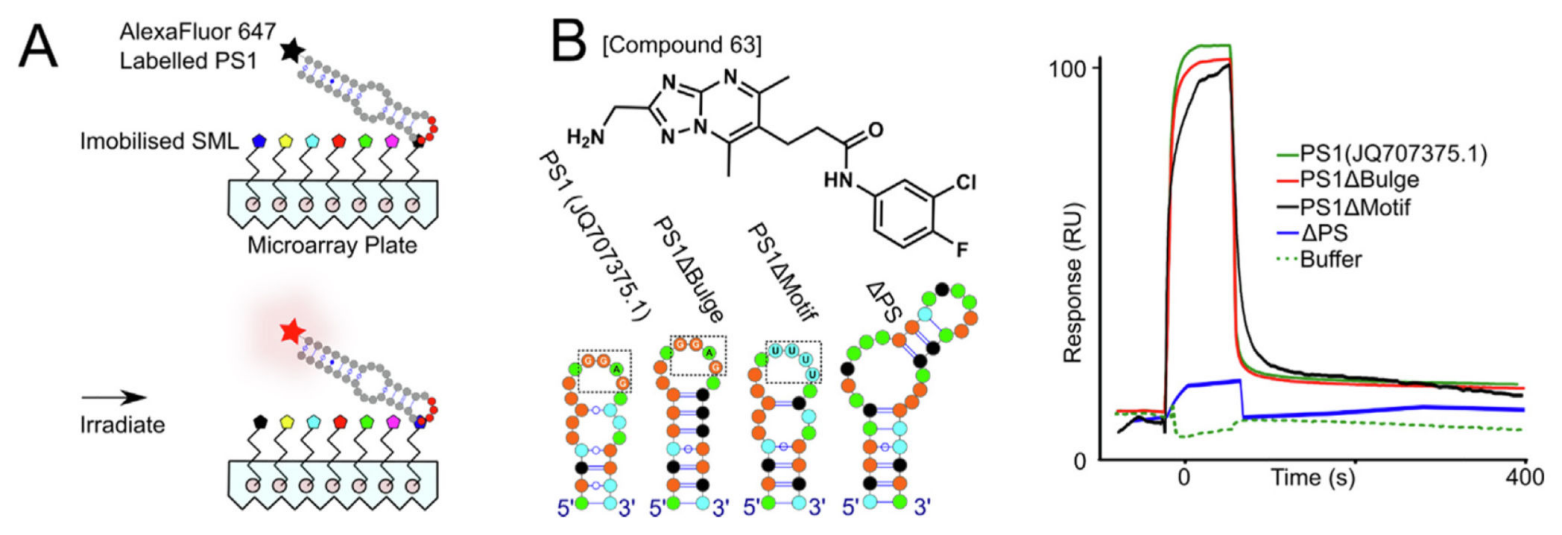
Identification of PS1-binding compounds and their PS affinities. (A) Cartoon of AlexaFluor 647-labelled (black star) PS1 RNA oligonucleotide binding to immobilized ligands in the SMM. The fluorescent signals identifying potential binders is shown in Sup Figure 3. (B) *Left, top,* Structure of Compound #63, together with the PS RNA oligonucleotides used to assess binding specificity (*Left, bottom*) - PS1; PS1ΔBulge; PS1ΔMotif & ΔPS stem loops. (Note nucleotides, here & throughout, are shown as coloured circles - green = A; orange = G; black = C; & cyan = U. Watson-Crick base pairs are indicated as lines, interrupted by circles for G-U pairs. Mfold folding energies of these RNA stem-loops are shown in Figure 3). *Right,* SPR traces for Compound #63 (40 nM) binding to immobilized PS variants: PS1 (green); PS1ΔBulge (red); PS1ΔMotif (black), and the unstructured (US) control (blue), Figure 3. Buffer alone SPR trace against PS1 (dashed green) is also shown. Compounds were washed over immobilized RNA oligonucleotides in a buffer containing 20 mM HEPES (pH 7.5), 250 mM NaCl, 2%(v/v) DMSO and 0.1%(v/v) Tween20, at a rate of 30 μL/min for 1 min at 37 °C, with the dissociation of compounds monitored for 5 min.

**Figure 3:**
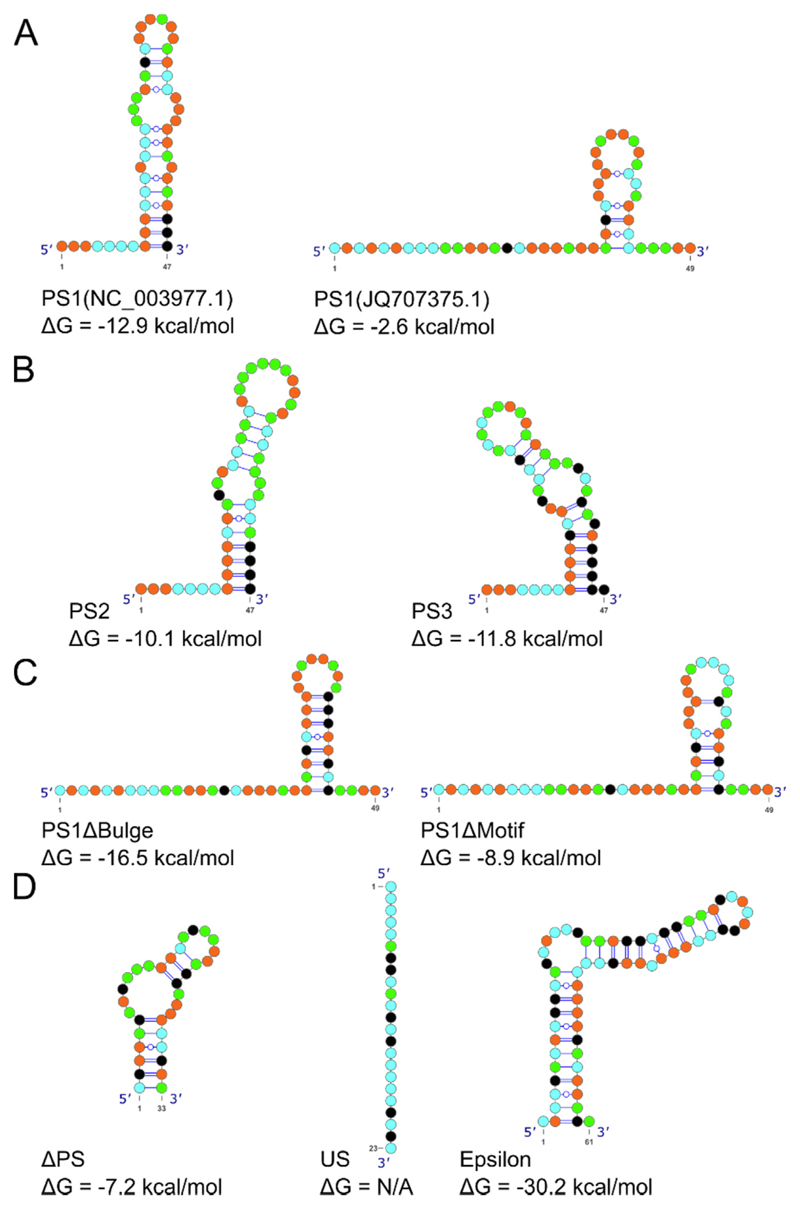
Sequences and secondary structures of RNA oligonucleotides used. (A) PS1 (NC003977.1 and JQ707375.1) oligonucleotides. G:C clamps were used to induce the formation of the bulged stem-loop structure in the sequences from the NC_003977.1 strain, presenting the Cp-recognition motif -RGAG- within the apical loop. (B) Similar stem-loops encompassing the PS2 and PS3 sites from strain NC_003977.1. G:C. (C) PSΔBulge and PSΔMotif variants of the homologous PS1 stem-loop in strain JQ707375.1.^36^ (D) Control RNA oligonucleotides: ΔPS (an RNA stem-loop from unrelated picornavirus); US, an unstructured 23-mer; and epsilon (ε), the stem-loop at the 5' end of the HBV gRNA. Free folding energies for all RNA oligomers are shown below each fold.

Sixty-six, non-immobilized versions of these PS1-binding compounds are commercially-available. These were purchased, resuspended in DMSO (to 10 mM), and stored frozen until diluted for binding assays. Each compound was numbered (1, 2, 3, etc.) based on their relative positions in the initial SMM array. SPR^49,50^ was used to determine the binding affinities of each ligand for a series of RNA oligonucleotides, including the PS homologues from both NC_003977.1 &JQ707375.1 (Figures 2B and 3); as well as sequence/structure variants. These latter include NC_003977.1 PS2 & PS3, bulge & loop variants of the JQ707375.1 PS1 (PSΔBulge and PSΔMotif, respectively), and an unrelated stem-loop (ΔPS) lacking an −RGAG-motif and the epsilon stem-loop (Figures 2 and 3). 5'-biotinylated RNAs encompassing these sequences were immobilized onto streptavidin-coated sensorchips and their affinities and stoichiometries for the test compounds determined.

The apparent association (*k_a_*) and dissociation (*k_d_*) rate constants were calculated from their sensorgrams (Methods). The best fits to the data are with the two-state binding model within the T200 Biacore Evaluation Software (Table 1, Figure 2, Sup Table 2 and Sup Figure 4). Most compounds possess higher affinities for the selection target, PS1, although roughly a third bind better to the PS2 or PS3 oligonucleotides. These results are consistent with the functional conservation of the PSs despite sequence/structural changes. The worst binders were compounds 27, 54 and 55, which have affinities in the millimolar range. All compounds bound preferentially to PSs 2 and 3 compared to negative controls, implying recognition of a common feature. Only 5 of the 66 compounds tested show any binding to the negative control RNAs with affinities in the high millimolar range (compounds 1, 4, 25, 49 and 50, data not shown). The affinities of the compounds (*K_D_*) for PS sites range from nanomolar to millimolar. The RU changes on binding are consistent with formation of complexes with a stoichiometry of 2:1 ligand: PS RNA for the highest affinity binders, consistent with the best-fitting model applied to analyze the data. Lower affinity binders appear to bind less specifically with apparent saturations ~5:1 ligand: PS RNA.

#### Inhibition of NCP assembly

To test whether ligand binding by the PSs inhibits assembly of the HBV NCP *in vitro,* we used an established assay with a genome length transcript gRNA (~3400 nt), based on strain JQ707375.1. This RNA lacks the terminal modifications but still re-assembles *in vitro* to nuclease-resistant, mostly *T* = 4 NCPs.^36^ At low nanomolar concentrations, assembly of NCPs is dependent on sequence-specific interactions between Cp and gRNA,^31,36^ i.e. it mimics *in vivo* assembly outcomes.^32,51^ The titration of Cp into this gRNA was automated,^36^ allowing low concentration reassembly reactions to be carried out in 96-well plates. The products were then pooled and concentrated before characterization. Importantly, for the ligands tested here their affinities for PS1 from strain NC_003977.1, used for ligand identification, and JQ707375.1, used for *in vitro* reassembly are very similar (Table 1), implying that these PSs share the molecular features bound by the smaller molecules.

**Figure 4:**
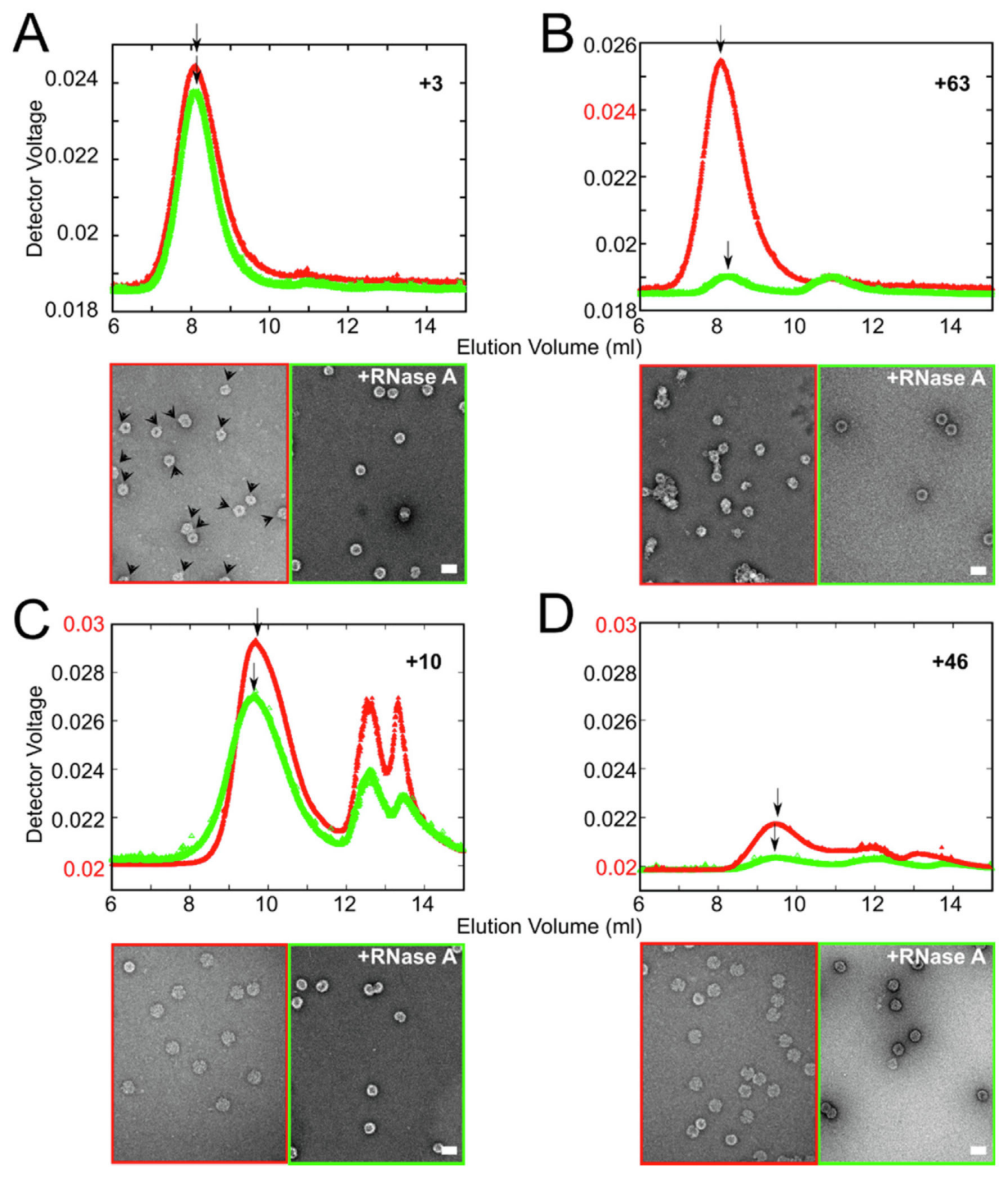
Inhibition of NCP reassembly by PS-binding compounds. *In vitro* reassembly reactions were set up using a liquid-handling robot to titrate Cp dimer (final concentration = 1.2 μM) into heat-annealed gRNA (1 nM) in a 96-well plate.^36^ Products were concentrated and analyzed, before (red) and after (green) RNase treatment by gel filtration (top traces) and nsEM (lower micrographs). Gel filtration light-scattering traces are shown for (A) Compound #3; (B) Compound #63; (C) Compound #10; & (D) Compound #46, each at a final concentration of 10 μM. R*_h_* values were recorded (Table 2) at the positions indicated by arrows. Scale bars, here and throughout = 50 nm.

To assess the inhibitory effects of PS-binding ligands, small-scale reactions (90 μL) at nanomolar concentrations were performed, titrating 1.2 μM Cp in 10 steps over a 2 h period into a solution of gRNA transcript (1.1 nM, final concentration 1 nM). Ligands were added (final concentration 10 μM, unless stated otherwise) to the gRNA solution prior to Cp titration, allowing the full range of ligand affinities to be tested. Pooled samples from 96-well plates were split into two aliquots. RNase A was added to one of these to degrade unencapsidated gRNA and then samples of each aliquot were visualized by negative-stain electron microscopy (nsEM) (Figure 4). The remainder of each aliquot was then fractionated by size-exclusion chromatography, monitoring the outflow via a multiple-angle, laser light-scattering detector (SEC-MALLS). These steps allow the integrity, shape, and hydrodynamic radius (R_*h*_) of assembled particles to be assessed, and they provide an estimate of assembly efficiency (Figure 4). Peaks eluting from the gel filtration column were re-concentrated to determine their RNA and Cp content via their corrected *A*_260/280_ ratios (Table 2). Note, DMSO at the concentration used here has no effect on reassembly. A dominant, single symmetrical light-scattering peak (red) elutes from the column at ~8-8.5 mLs post-injection. These NCPs have a R*_h_* of 18.2 nm (Table 2), slightly smaller than that of NCPs produced by Cp expression in *E.coli* in the absence of gRNA (19.4 nm).^36^ Their indifference to nuclease treatment (Figure 4, Table 2) implies that the assembled Cp shells are complete.

Compound #3 has one of the lowest affinities for PS1 (*K_D_* ~ 210 μM) and binds PS3 poorly. However, it binds PS2 reasonably tightly (*K_D_* μ 300 nM), implying that this PS will be fully saturated at the test concentration. Reassembly in the presence of Compound #3 [10 μM] reveals the formation of largely nuclease-resistant NCPs (Figure 4A) eluting in the same position as those from ligand-free controls. Note, the filter used to concentrate samples removes unassembled Cp from the traces. The *A*_260/280_ ratio of this peak is also similar to the control, implying that Compound #3 has very little effect on NCP assembly, consistent with reassembly in the presence of gRNA transcripts lacking PS3.^36^ However, the yield of NCP is reduced (>5-fold) in the presence of Compound #3 compared to the uninhibited control. This level of variation is striking (Table 2) and cannot be accounted for unless the ligand behaves as an assembly inhibitor. EM images offer support for this conclusion. Prior to nuclease treatment, most reassembled particles lack the smooth circular appearance of the uninhibited control, and stain penetration is variable. In addition, many particles appear to have a unique, bright structural feature on their peripheries (see arrowheads on the micrograph, Figure 4A). These mostly disappear with nuclease treatment implying that they are due to incompletely protected gRNA.

Although the impact of Compound #3 appears limited, this result shows that it partially inhibits correct encapsidation, consistent with the drop in NCP yield.

Compound #63 has the highest affinity for PS1 (*K_D_* ~ 19 nM), the most important PS for regulation of *in vitro* NCP formation.^36^ Consistent with this, reassembly in the presence of this ligand shows the strongest inhibitory effects. Prior to nuclease treatment the NCPs formed have the same apparent *R_h_* as the control, with a yield similar to that obtained with Compound #3. However, nuclease treatment degrades the majority of the NCPs formed, leaving only minor peaks on the gel filtration profile. EMs of the NCPs formed in the presence of Compound #63 are strikingly different than the control. Post-RNase treatment, most particles are heterogeneous and heavily stain-penetrated, and have formed large clumps with relatively few remaining as separate shells of the correct size (Figure 4B). Absorption values confirm these interpretations (Table 2). Similar inhibitory effects were also observed with Compounds #10 and #46, both of which have intermediate affinities for PS sites (Table 1, Figure 4C and D). Both compounds result in low yields of nuclease-sensitive, particulate material that elutes later than the NCP peak in the uninhibited control. In each case there are multiple additional peaks, implying that assembly is significantly dysregulated by these PS-binding compounds.

**Figure 5:**
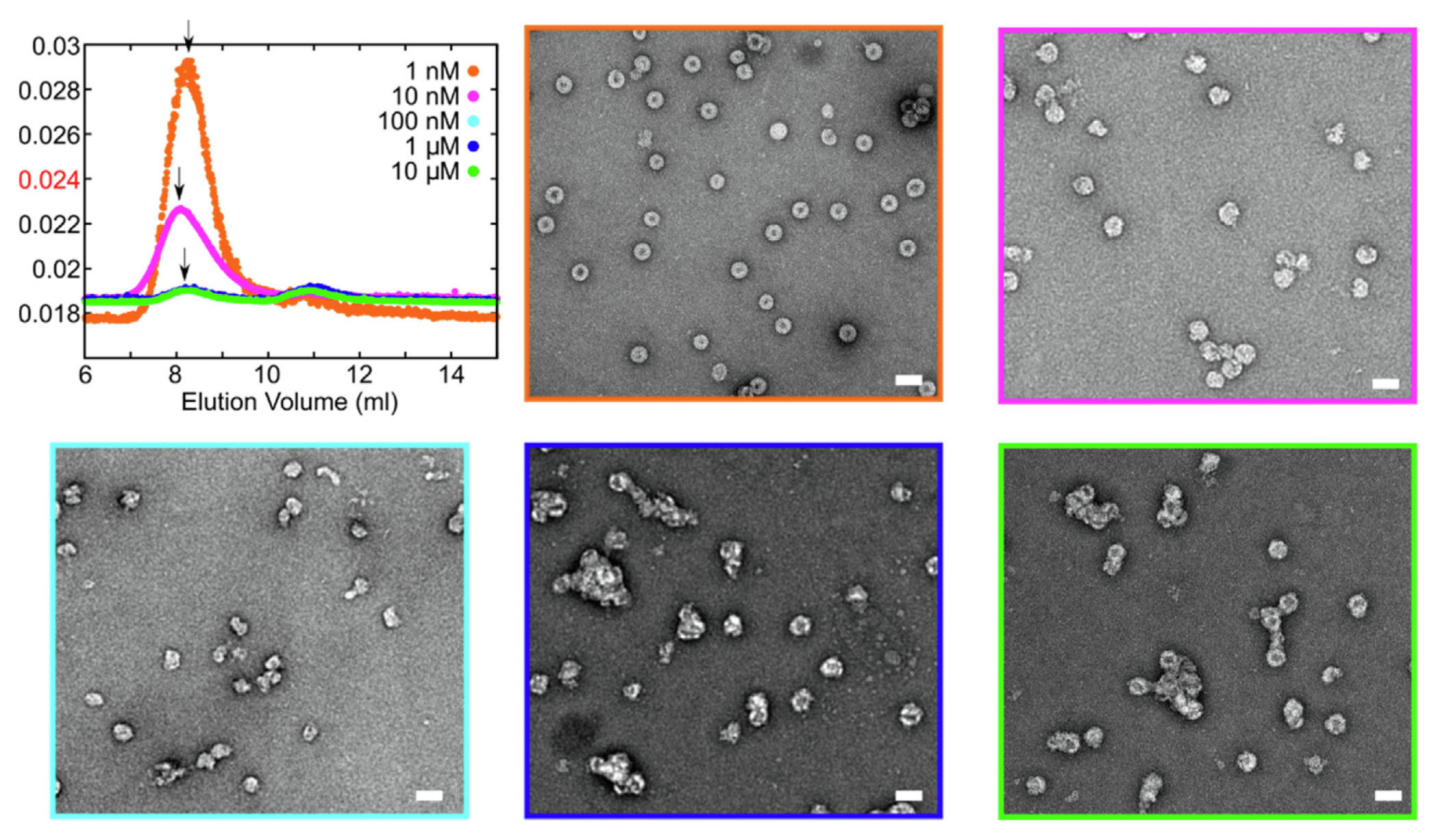
Concentration dependence of assembly inhibition by Compound #63. (A) LS traces of the NCP reassemblies at differing Compound #63 concentrations (details as in Figure 4): 1 nM (orange), 10 nM (magenta), 100 nM (cyan), 1 μM (blue) and 10 μM (green) prior to RNase treatment. Colour-coded nsEMs are shown. Nuclease treatment yields particulate products similar to reassembly in the absence of gRNA (Sup Figure 7).

To explore the concentration dependence of these effects the final concentration of Compound #63 was serially diluted 10-fold to a final concentration of 1 nM, i.e. until it was equimolar with the gRNA assembly substrate (Figure 5; Table 3). Nuclease-resistant NCP yields increase from ~15% at the highest ligand concentration to ~90% of the control value at the lowest concentration. Even at 1 nM ligand concentration, i.e. well below the apparent affinities for all three PSs (Table 1), some assembly dysregulation occurs. Reductions in NCP yield are accompanied by significant changes in the appearance of the samples in nsEM (Figure 5). At the lowest ligand concentration (1 nM) particles are largely separate with circular cross-sections, although most of these show stain-penetration with some small variations in apparent diameter. At higher ligand concentrations, they become much less regular and start to clump together, and this tendency increases with the level of assembly inhibition. Binding of Compound #63 to the RNA PSs, which the SPR data suggests occurs at all three sites, appears to be the direct cause of these effects. Since clumping occurs it suggests that even when reassembled at low concentration there is a tendency for Cp to bind additional gRNAs. That would be consistent with the appearance of separate particles after nuclease treatment.

#### Structure activity relationship (SAR) of Compound #63

**Figure 6:**
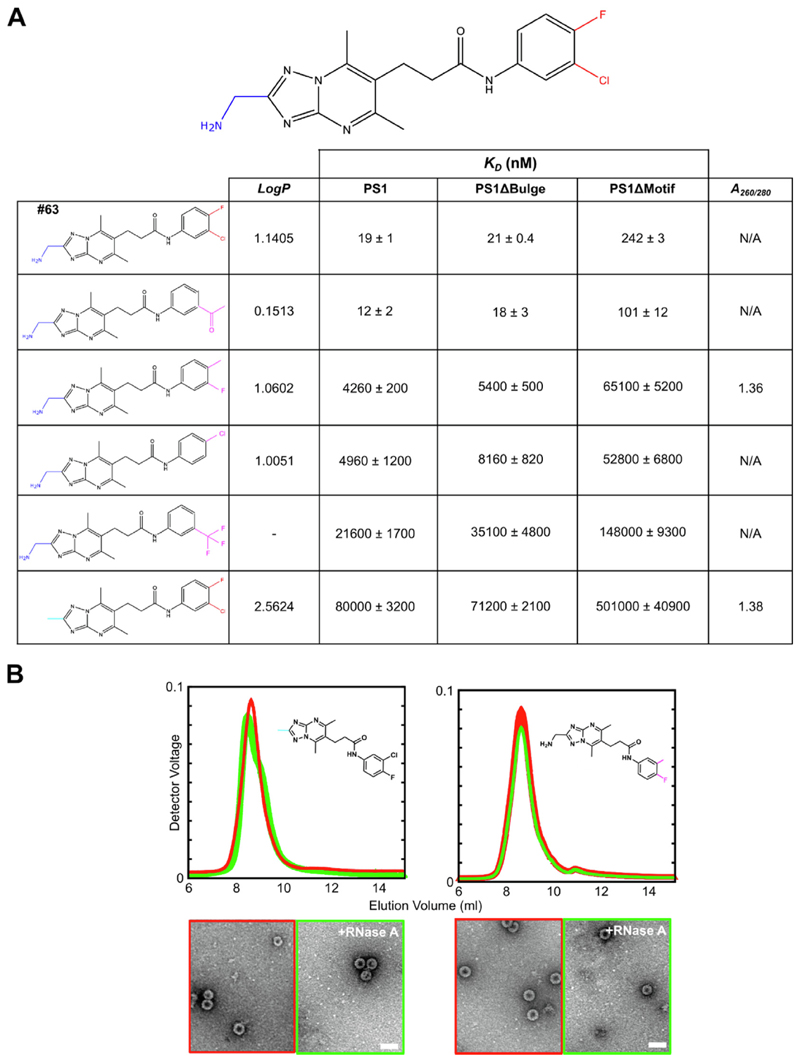
SAR analysis of Compound #63. (A) Compound #63 variants at the pendent amino and halogen functional groups (blue and red, top) were varied (cyan and magenta, respectively). SPR was used to determine their affinities (*K_D_*) for PS1 and its two variants, lacking the bulge in the stem (PS1ΔBulge) or the Cp-recognition motif in the loop (PS1ΔMotif). Where available, the logP value for each compound is shown, right. (B) Two compounds (structures inset) with low affinities for PS1 were used in NCP reassemblies, at a final concentration of 10 μM (details as in Figure 4). The *A*_260/280_ ratios and ns-EMs of the NCPs formed are shown in (A), right.

The PS-binding ligands possess many different chemophores. To understand which structural features of Compound #63 contribute to its ability to bind PS sites tightly, a structure—activity relationship (SAR) of its pendent functional groups was carried out (Figure 6, Sup Figure 5). Five commercial ligand variants were used to explore the role(s) of the halogens in the C6 ring and the amino group on the C5 ring. There is a >4000-fold drop in affinity for PS1 upon removal of the latter, highlighting it as a key feature for RNA recognition. Removal of both halogens appears fully compensated by inclusion of an aldehyde instead of chorine. All other variants that including swapping the positions of chlorine and fluorine, or the substitutions and removals, cause 200–1000-fold drops in PS1 affinity. Binding of these ligand variants to JQ707375.1 PS1 lacking a bulged stem (PS1ΔBulge) (Figure 2) seem only modestly affected by these ligand substitutions (Figure 6). In contrast, removal of the Cp-recognition loop motif (PS1ΔMotif) leads to significant drops in affinity. These results suggest that the primary ligand binding site is the Cp-recognition motif in the stem-loop.

These experiments suggest that the PS-binding ligands inhibit Cp-binding to HBV gRNA, and thus NCP assembly regulation. We therefore explored whether these compounds would inhibit HBV infections in cell culture. Although we did see examples of such effects with some of the ligands (Dorner, *pers. comm*.), they were not reproducible at concentrations up to 100 μM. This is surprising given the known *logP* values of the compounds (Table 1).^52,53^ However, by preventing the formation of NCPs *in vitro,* the ligands identified here possess the properties required for a directly-acting antiviral (DAA) for HBV. In addition since they target multiple sites across the pgRNA. Since these are highly unlikely to undergo simultaneous mutation and also relatively conserved across strain variants, they represent a stable and novel molecular target.^54^ The preliminary SAR data reported above can be used by synthetic groups to identify inhibitors with appropriate *in vivo* characteristics. In order to facilitate such studies the *in vitro* NCP assembly assay has been adapted to a semi-high-throughput, multi-well plate format. This assay monitors changes to the anisotropy of dye-labelled PS-encompassing oligonucleotides during assembly (Sup Figure 6).

#### Modelling the anti-viral effects of PS-binding compounds

Given the urgent clinical need for additional HBV inhibitors, we also explored the effects of PS binding ligands on HBV replication using an intracellular computational model. This is useful because there are very few susceptible organisms for HBV infection, other than primates. Ethical considerations therefore inhibit widespread testing of synergic effects between potential DAAs, whereas these can be assessed readily in the mathematical model.

The model used^44^ describes different aspects of the viral life cycle in the context of several discrete reactions (Sup Figure 1), describing processes from viral entry to envelopment, thus facilitating the comparison of different antiviral strategies. Previously, virion release kinetics from a single infected cell were modelled both in the absence of therapy, and in the presence of different antiviral strategies, including the PS-targeting compounds analysed here. These compounds were^44^ comparable in performance to other assembly inhibitors, such as the Cp allostery modulators (CAMs), currently in clinical trials in chronic HBV patients.^55^

**Figure 7:**
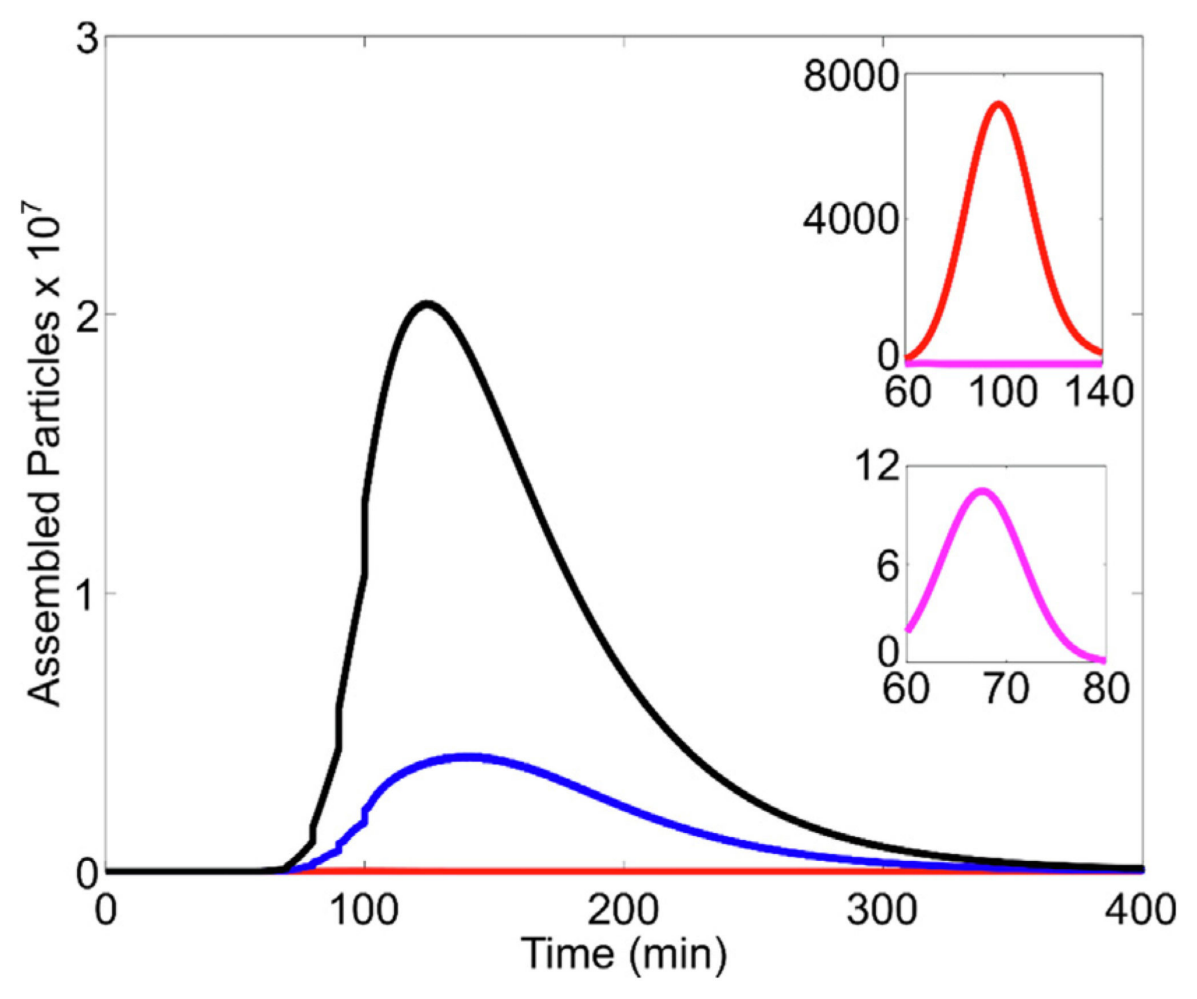
Modelled NCP assembly in the presence of Compound #63. The numbers of fully-formed particles at 160 min post assembly initiation, calculated from a Gillespie simulation, are plotted against time for differing concentrations (0 (black), 1 (blue), 5 (red) & 10 (magenta) nM, respectively) of Compound #63.

Here, we focus on modelling the impacts of the assembly inhibition observed *in vitro* on this full cellular model, (Sup Figure 1, green highlights) enabling direct comparison of the effects of various compounds. The outcomes of this reduced model, directly inform the full cellular model, as well as within host models of a viral infection^56^ that also take the host immune response into account. The reaction kinetics of NCP assembly around gRNA were modelled^44,57^ to mimic the conditions used in the titration assay described here (see Methods). Figure 7 shows the predicted time course of intact particle formation, without ligand (black) and for different concentrations of Compound #63 (1 nM, blue; 5 nM, red; 10 nM magenta). In the absence of inhibitory ligands, the number of complete particles 160 min post-initiation is 7.2 × 10. This decreases to 1.4 × 10^10^, 1.5 × 10^7^, and 6.4 × 10^3^ in the presence of 1, 5 and 10 nM of Compound #63, respectively.

A comparative analysis of the effects of the other compounds, at concentrations of 1, 3 and 30 nM was also performed, assuming a gRNA concentration of 1 nM (Table 4). For Compounds #3, #10 and #46, when going from 1 to 30 nM ligand we see modest drops in particle numbers of 2, 5 and 4 × 10^10^, respectively. However, at 30 nM, Compound #63 is predicted to inhibit particle formation completely (Table 4).

## Discussion

As expected for PS-mediated assembly regulation,^31,36^ individual PSs vary in their importance for regulation of HBV NCP assembly *in vitro*.^35^ PS1 is the major regulatory requirement for assembly around the genome length ssRNA transcript used here. Disruption of the PS1 Cp-recognition site by mutation of 6/3400 nts leads to severe inhibition of NCP assembly *in vitro.*^36^ High-affinity PS1 sequence binding ligands, are also potent assembly inhibitors of NCPs *in vitro,* opening up a novel antiviral target.^44^

These data are the first example of DAAs targeting HBV PS-mediated NCP assembly. Other DAA treatments for HBV infection are being developed, such as CAMs. CAMs have been shown to disrupt HBV NC assembly at micromolar concentrations, and are based on the initial discovery of heteroaryldihydropyrimidine (HAP) compounds.^58–62^ These compounds show early promise in clinical trials^60,63–66^ and it will be interesting to see if the potential synergy with the anti-PS-mediated DAAs probed here by modelling can be made to work *in vivo* in cells. Development of drugs targeting multiple distinct steps of the HBV life cycle may be required to achieve an effective cure, as it has been with HIV.^67^ The mathematical modelling of infection is important in such cases because it allows exploration of conditions that might be difficult to achieve experimentally.

There are currently few clinical drugs that target RNA. Risdiplam, a small molecule targeting RNA for treatment of spinal muscular atrophy, has however recently gained FDA approval.^68^ This development is an important milestone, since the human transcriptome is considerably larger than the proteome, which contains most current drug targets. Targeting RNA with oligonucleotides is established but using small ligands is advantageous because of their easy administration.^69^ Previously, anti-HIV ligands that prevent its assembly by binding to the assembly initiation signal in its gRNA were isolated, and shown to inhibit gRNA incorporation.^70,71^

Here we show that it is possible to dysregulate HBV NC assembly *in vitro* using drug-like ligands that bind to the RNA PSs of its pgRNA. The best of these inhibitors binds with nanomolar affinity and SAR (Figure 6) suggests that these are good starting points for further development of directly-acting anti-viral drugs (DAAs). The logP values of these ligands are such that they should gain access to the cytoplasm, where NC assembly occurs, with ease. Understanding why the *in vitro* inhibitory effects are absent in cell culture is not trivial. Nevertheless, the data presented here describe the first example of isolation and characterisation of ligands and their effects *in vitro,* for an assembly regulatory element, i.e., the PSs of the HBV pgRNA.

Since we^28,29^ and others^72^ have shown that ssRNA viruses, including major human pathogens in the picorna- and alphaviruses mostly lacking antivirals or vaccines,^28–34^ also use this form of assembly regulation, the approach described here to identify novel DAAs could be useful. The ease of the selection of these ligands is proof of principle that potential DAAs for virions that use PS-mediated assembly regulation could be rapidly identified. Comparison of strain variants in these cases suggests that the multiple PSs regulating virus assembly are highly conserved. As drug targets this makes them resilient to error-prone genome replication.

## Supplementary Material

Supplementary data to this article can be found online at https://doi.org/10.1016/j.jmb.2022.167557.

Supplementary Material

## Figures and Tables

**Table 1:** PS affinities & LogP values of test compounds.

Compound	K*_D_*/nM			LogP
	PS1(NC_003977.1)	PS1 (JQ707375.1)	PS3	
3	211,000 ± 1179	224,000 ± 455	99,100 ± 591	4.0467
10	11,800 ± 284	11,400 ± 170	8530 ± 797	3
46	10,100 ± 119	10,200 ± 225	32,700 ± 227	1.32
63	12 ± 0.3	19 ± 0.5	15 ± 0.01	1.1405

Left, Binding affinities (nM) and their associated standard error of the mean of PS binding compounds characterised in this study, given to 3 significant figures, for RNA oligonucleotides encompassing PS1 from different strains of HBV (Left to right, NC_003977.1, JQ707375.1) and PS3. Right, log P values for each PS binding compounds. Full table available in Supplementary Information (Sup Table 2).

**Table 2:** Effects of ligands on gRNA-mediated NCP reassembly in vitro.

Compound	NCP Peak		
	*A*_260/280_	RNA yield	R*_h_*/nm
N/A	1.43	92%	18.2
3	1.42	67%	18.0
10	1.23	63%	15.2
46	0.76	33%	14.7
63	0.98	16%	17.2

Left to Right *A*_260/280_ ratios, packaged RNA yield, and R*_h_* values for reassembled materials resulting from the interaction between Cp and gRNA, in the absence and presence of Compounds #3, 10, 46 and 63. Values are shown after treatment with RNase A.

**Table 3:** Concentration-dependence of NCP assembly Inhibition by Compound #63.

Compound #63/nM	NCP Peak
	*A*_260/280_	RNA yield	R*_h_*/nm
1	1.49	90%	17.7
10	1.46	46%	18.3
100	1.03	15%	16.4
1000	0.86	8%	18.1
10,000	0.93	15%	18.6

The yields and appearance of the NCPs produced at differing ligand concentrations (1, 10, 100, 1000 and 10,000 nM Compound #63) are listed above. Values are shown after nuclease treatment.

**Table 4:** Computational modelling of assembly inhibition by PS-binding ligands.

Compound	Concentration			
	1 nM	3 nM		30 nM
3	7.1 × 10^10^	6.9 × 10^10^		5.1 × 10^10^
10	6.2 × 10^10^	4.7 × 10^10^		1.0 × 10^10^
46	6.6 × 10^10^	5.8 × 10^10^		2.1 × 10^10^
63	1.4 × 10^10^	6.0 × 10^8^		0.0

## Data Availability

Data will be made available on request.

## Data availability

Data available upon request. Requests should be directed towards NP or PGS.
