# Supplementary material for: Dysregulation of Hepatitis B Virus Nucleocapsid Assembly in vitro by RNA-binding Small Ligands
Source: J Mol Biol. Author manuscript; Available in PMC 2022 Apr 21. (PMC7612645; doi:10.1016/j.jmb.2022.167557)
Supplement: Supplementary Material [file EMS144476-supplement-Supplementary_Material.pdf]

Supplementary Information for Dysregulation of Hepatitis B Virus Nucleocapsid Assembly *in vitro* by RNA-directed Small Ligands

Nikesh Patel[1◊], Fardokht Abulwerdi[2*], Farzad Fatehi[3], Iain Manfield[1], Stuart Le Grice[2], John S. Schneekloth, Jr.[2], Reidun Twarock[3] & Peter G. Stockley[1◊]

[1]Astbury Centre for Structural Molecular Biology, University of Leeds, Leeds, LS2 9JT, UK.

[2]Center for Cancer Research, National Cancer Institute, Frederick, MD 21702-1201

[3]Departments of Biology and Mathematics & York Centre for Complex Systems Analysis, University of York, York, YO10 5DD, UK

[◊]Joint communicating authors

*Current Address: Center for Drug Evaluation and Research (CDER), US Food And Drug Administration, Washington DC, USA

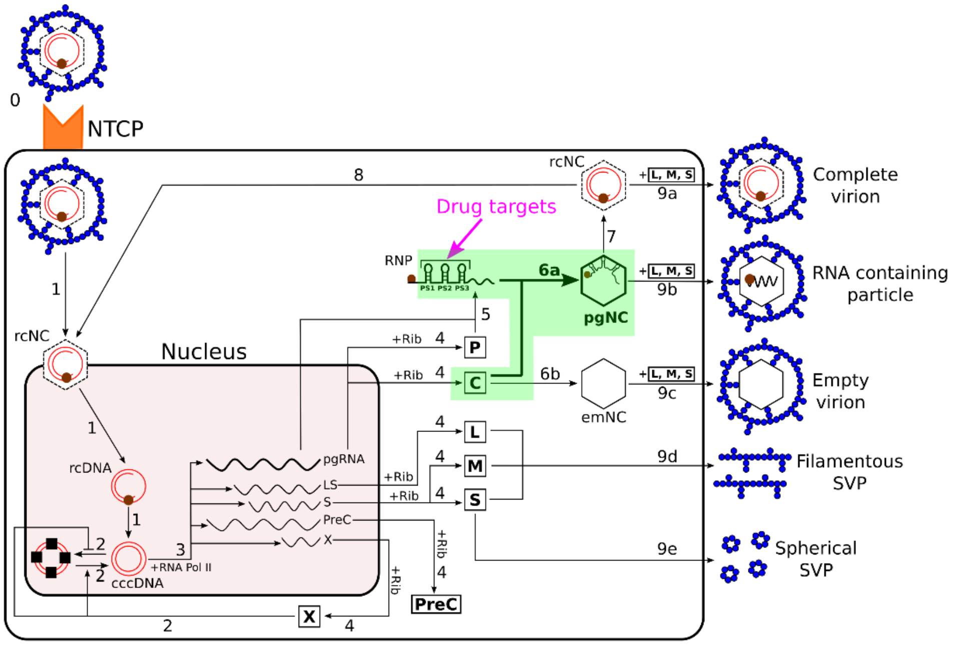

Supplementary Figure 1: The hepatitis B virus (HBV) life cycle (courtesy of [1]***).***

The assembly stage of HBV nucleocapsids is highlighted green. (0) Viral entry is mediated by the sodium taurocholate co-transporting polypeptide (NTCP) receptor, which allows the nucleocapsid (rcNC) containing relaxed circular DNA (rcDNA) to be released into the cell via endocytosis (not shown). (1) After attachment to the nucleus, the rcDNA is delivered. Host DNA repair factors convert the rcDNA into covalently closed circular DNA (cccDNA). (2) X protein blocks the silencing of cccDNA, allowing for transcription. (3) RNA pol II transcribes viral HBV RNAs, including the pgRNA; LS, S, PreC, and X mRNAs. The pgRNA encodes both the core (C) and polymerase (P) proteins; X mRNA the X protein; the LS and S mRNAs encode the L, M and S surface proteins; and PreC mRNA the precore (PreC) protein. (4) mRNAs are translated by ribosomes (Rib). (5) The pgRNA and P form a 1:1 assembly competent ribonucleoprotein (RNP) complex. (6a) Encapsidation of the RNP complex by C proteins to form a nucleocapsid containing pgRNA:P (pgNC). (6b) Assembly of C proteins into an empty nucleocapsid (emNC). (7) The reverse transcription of pgRNA by P within the pgNC resulting in the conversion of pgNC into an rcDNA-containing nucleocapsid (rcNC), which is also termed

the mature nucleocapsid. (8) rcDNA is recycled from some mature nucleocapsids to form more cccDNAs. (9a) Envelopment of mature nucleocapsids within a membrane layer containing the surface proteins L, M, and S, allowing for the secretion of a complete virion. (9b) Some RNA containing immature nucleocapsids can be enveloped, resulting in secretion of an RNA-containing particle. (9c) Envelopment of an empty nucleocapsid and secretion of an empty virion. (9d) L, M, and S proteins can form empty filaments and filamentous subviral particles (SVPs) via conventional tubular budding into late endosomes and exit the cell. (9e): S proteins assemble into octahedral spheres (spherical SVP) and exit the cell.

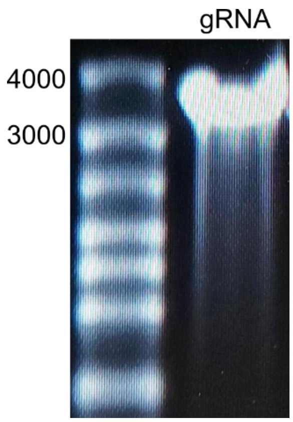

Supplementary Figure 2: Analysis of in vitro transcribed gRNA.

(C) *In vitro* transcribed gRNA, was separated on a denaturing 1% (w/v) agarose gel and visualised by staining with EtBr. *Left,* RiboRuler High Range RNA Ladder (ThermoFisher Scientific), sizes shown in nt. *Right,* gRNA.

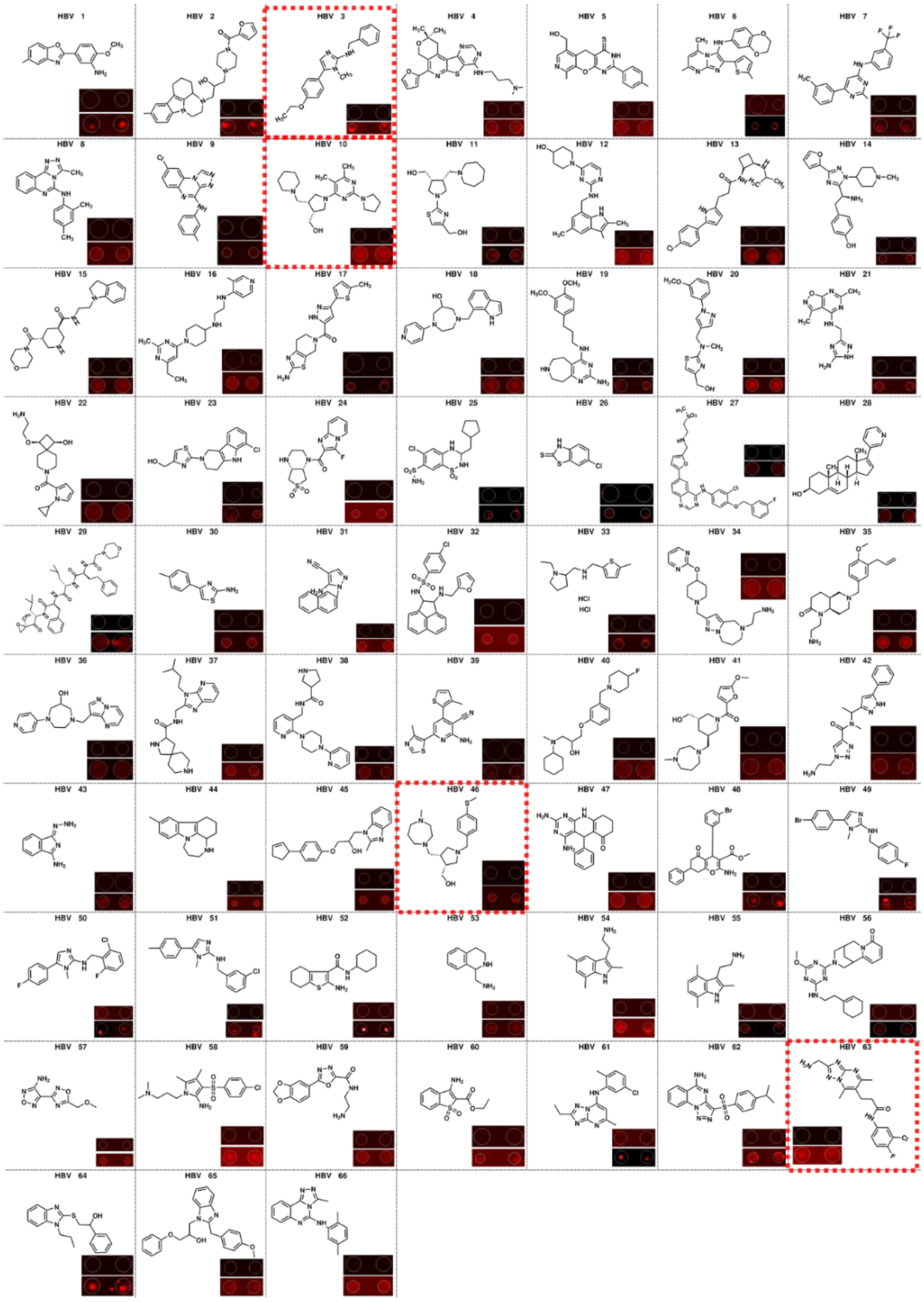

Supplementary Figure 3: PS1 binding compounds.

These 66 compounds shown are the commercially available subset of the 72 immobilized ligands in the SMM that showed red fluorescence (bottom panels) above the background (top panels) when washed with an AlexaFluor 647-labelled oligonucleotide encompassing annealed PS1 or buffer (RNase-free 1× PBS (12 mM NaH_2PO_4, 137 mM NaCl, 3 mM KCl, pH 7.4)) alone.

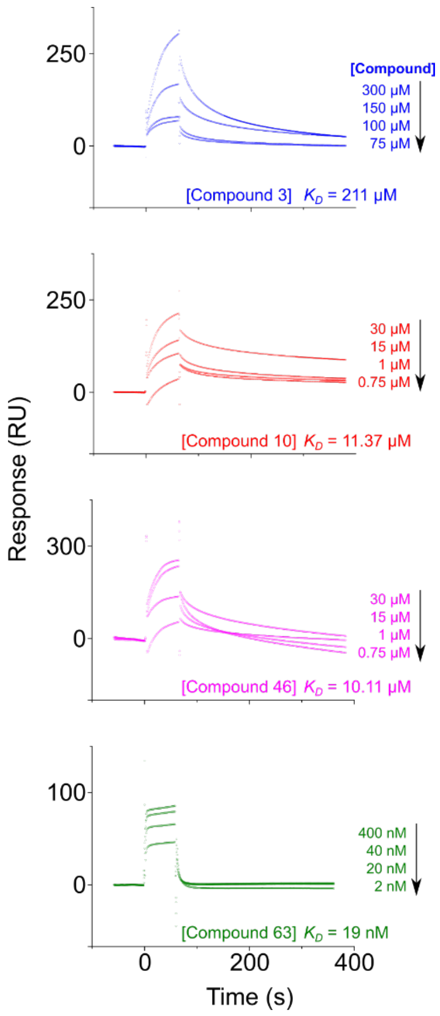

Supplementary Figure 4: SPR Traces for hit compounds.

Top to bottom, Raw SPR traces for Compound #3 (blue), #10 (red), #46 (magenta) and #63 (green) binding to immobilized PS1(NC_003977.1): Fits from titrations, detailed right, are shown. Measured K_D values are shown right. Compounds were washed over immobilized RNA oligonucleotides in a buffer containing 20 mM HEPES (pH 7.5), 250 mM NaCl, 2%(v/v) DMSO and 0.1%(v/v) Tween20, at a rate of 10 μL/min for 6 min at 37 ^{o}C.

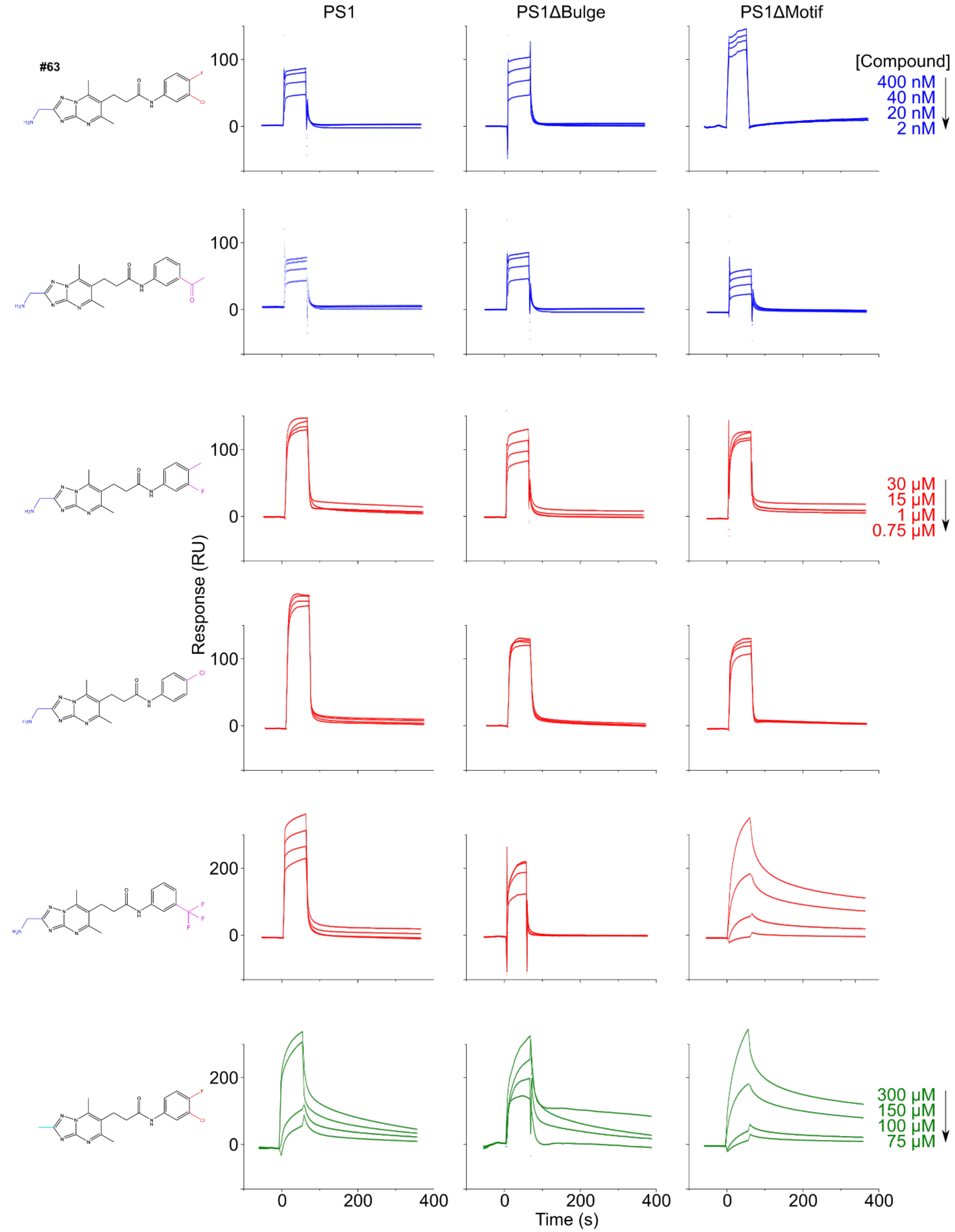

#63
PS1
PS1ΔBulge
PS1ΔMotif
[Compound]
400 nM
40 nM
20 nM
2 nM
30 μM
15 μM
1 μM
0.75 μM
300 μM
150 μM
100 μM
75 μM
Response (RU)
Time (s)

Supplementary Figure 5: SPR Traces for SAR investigation.

Top to bottom, Raw SPR traces for Compounds shown in figure 6 (*one compound per row – compounds shown left*) binding to immobilized PS1(JQ707375.1), PS1ΔBulge and PS1ΔMotif (*columns from left to right*): Fits from titrations, detailed right, are shown. Measured K_D values are shown in figure 6. Compounds were washed over immobilized RNA oligonucleotides in a buffer containing 20 mM HEPES (pH 7.5), 250 mM NaCl, 2%(v/v) DMSO and 0.1%(v/v) Tween20, at a rate of 10 µL/min for 6 min at 37 ^{o}C.

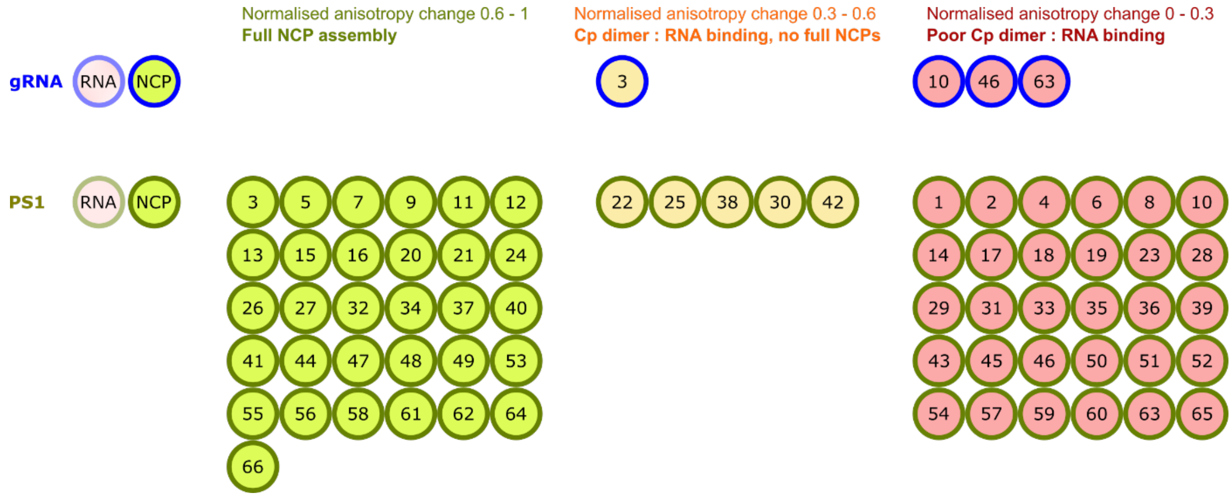

Supplementary Figure 6: Summary of plate-based assembly inhibition assays.

Heat annealed 1 nM gRNA (top) or 15 nM PS1 (bottom) oligo (NC_003977.1, Fig 3) were dispensed into a 96-well plate (blue, green outlines respectively). 10 µM compound were added to wells as labelled. 1.2 µM Cp dimer was titrated as described in Methods [2] and any assembled material then challenged with 1 µM RNase A. The final normalised fluorescence anisotropy was calculated, and control reactions ± Cp used to denote full NCP (1) and degraded RNA (0).

Anisotropy change is displayed here by the colour fill in each well. Left to right: *light green* 0.6-1, suggestive of efficient NCP assembly; *light yellow* = 0.3-0.6, suggestive of some Cp dimer: RNA binding; and *light red* = 0-0.3, suggestive of poor Cp dimer: RNA binding.

Assay was performed in triplicate, and the average anisotropy change shown, full data with associated standard error of the mean available in Supplementary Tables 4 and 5, for PS1 and gRNA reassemblies, respectively.

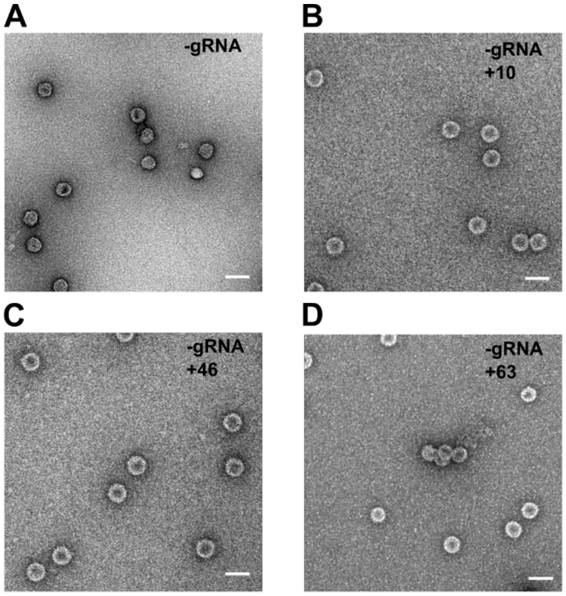

Supplementary Figure 7: Cp self-association is unaffected by PS-binding compounds.

(A) Self-assembly of Cp dimer in the absence of ligands; (B) as in (A) with 10 μM Compound #10 added; (C) as in (B) with 10 μM Compound #46; and (D) as in (B) with 10 μM Compound #63. Negatively stained images of these assembly mixes.

RNA Oligonucleotide	Sequence 5' → 3'
PS1(NC_003977.1)	GGGUUUUGGGUUUGUUUAAAGACUGGGAGGAGUUGGGGGAGGAGCCC
PS1(JQ707375.1)	UGUGUGUUUAAGGACUGGGAGGAGCUGGGGGAGGAGAUUAGGUUAAAGG
PS1ΔBulge	UGUGUGUUUAAGGACUGGGAGGAGCUGGGGGAGGAGACCCGGCUAAGG
PS1ΔMotif	UGUGUGUUUAAGGACUGGGAGGAGCUGGGGGAUUUUACUAGGCUCAAGG
PS2	GGGUUUUGGGGUGACAGUUAAUGAAAAAAGGAGAUUAAAAUUACCCC
PS3	GGGUUUUGGGGCUGGCAUUCUAUAUAAGAGAGAAACUACACGCCCCC
ΔPS	UCGGACAGCAAAGGUACAAGGACCAGGGUUCGA
US	UUUUUACCUAUCUCUUUUUCUCU
Epsilon	UGUUCAUGUCCUACUGUUCAAGCCUCCAAGCUGUGCCUUGGGUGGCUUUGGGGCAUGGAC<br>A

Supplementary Table 1. RNA Oligonucleotides used in SPR binding assays.

	Affinities / nM			
Compound	PS1 (NC_003977.1)	PS1 (JQ707375.1)	PS2	PS3
1	165 ± 2		141 ± 4	1750 ± 125
2	10000 ± 119		86400 ± 101	44100 ± 28
3	212000 ± 1179	224000± 455	320 ± 5	99100 ± 591
4	171000 ± 24900		1910 ± 83	59200 ± 3220
5	239 ± 1		50300 ± 1890	312 ± 1
6	61 ± 2		112 ± 1	615 ± 22
7	456 ± 5		174000 ± 248	491 ± 5
8	31 ± 2	92 ± 12	385000 ± 1040	1720 ± 1
9	30 ± 10		2890 ± 45	37 ± 3
10	11800 ± 284	11400 ± 169	10800 ± 691	8530 ± 797
11	11400 ± 220		147 ± 35	27100 ± 1234
12	61800 ± 4538		46300 ± 271	94700 ± 8466
13	20100 ± 20		26200 ± 375	206 ± 17
14	345000 ± 22		65000 ± 167	161000 ± 1010
15	32 ± 3		6 ± 4	1 ± 2
16	18300 ± 92		22900 ± 135	21500 ± 159
17	135	421 ± 54	199000 ± 402	9960000 ± 2000
18	83500 ± 700		124000 ± 5250	58500 ± 2180
19	19300 ± 900	12100 ± 231	16800 ± 565	16820 ± 1140
20	11 ± 5		66000 ± 3479	2280 ± 23
21	20 ± 2		30300 ± 79	3930 ± 526
22	18500 ± 264		37800 ± 29	932000 ± 4200
23	429000 ± 50		31 ± 6	368 ± 3
24	15 ± 4		14300 ± 20	44 ± 7
25	732 ± 57	1240 ± 87	42200 ± 29	12 ± 4
26	66 ± 9		2760 ± 2	52
27	816 ± 12		3960000 ± 7012	50700000 ± 1330
28	883 ± 4		57 ± 3	677 ± 8
29	16 ± 8	28 ± 9	182 ± 2	73 ± 17
30	177		169000 ± 8600	11 ± 1
31	154000 ± 6999		65400 ± 191	18 ± 1
32	64300 ± 32		547000 ± 4680	36
33	751000 ± 186		113000 ± 1950	1750
34	374000 ± 1500		116000 ± 3280	60400 ± 1600
35	1220 ± 150	1940 ± 65	348 ± 51	4730 ± 378
36	585 ± 37		239 ± 2	358000 ± 1000
37	37 ± 2		258000 ± 1350	3740 ± 3
38	55 ± 2	184 ± 31	124	1 ± 5
39	3 ± 7		467 ± 2	4540 ± 328
40	832000 ± 2750		58000 ± 7090	22700 ± 2500
41	532 ± 12		241000 ± 1380	697000 ± 53
42	18 ± 1		2090 ± 1	388 ± 32
43	17 ± 1		74	19 ± 1
44	519000 ± 546		173 ± 27	589000 ± 14600
45	248000 ± 371		17100 ± 798	162 ± 3
46	10100 ± 119	10200 ± 224	16500 ± 336	32700 ± 227
47	2		99 ± 2	73100 ± 4740
48	384 ± 5		112000 ± 572	90 ± 7
49	256 ± 24		83800 ± 1840	311000 ± 432
50	7 ± 3		170 ± 2	1240000 ± 850
51	133 ± 9		403000 ± 63500	336000 ± 100
52	7 ± 6		4660000 ± 7960	9 ± 2
53	751 ± 12	621 ± 56	299000 ± 7970	935 ± 10
54	37900 ± 189		7120 ± 1970	3590000 ± 26
55	24200 ± 1071		456000 ± 7680	1160000 ± 3233
56	1310 ± 3	768 ± 98	11 ± 2	57 ± 7
57	9 ± 5		227 ± 1	20 ± 11
58	23400 ± 243		13600 ± 8190	24600 ± 2230
59	0.1 ± 2		69 ± 1	29 ± 3
60	5 ± 3		49 ± 5	59300 ± 620
61	2 ± 4		33900 ± 232	217000 ± 1400
62	2000 ± 234		516000 ± 22700	4610 ± 392
63	12	19	20 ± 1	15
64	10 ± 7		46	79 ± 3
65	14		1	28 ± 4
66	272 ± 6		100 ± 1	2

Supplementary Table 2: SPR binding affinities (nM) of PS binding compounds for RNA oligonucleotides PS1, PS2 and PS3.

Left to right: Binding affinities (nM) and associated standard errors of the mean of the interaction between PS1(NC_003977.1)/PS1(JQ707375.1)/PS2/PS3 RNA oligonucleotides and compounds identified in the SMM. Errors <1 are omitted. Sensorgrams at 5 different compound concentrations were performed in triplicate. Control oligonucleotides show no significant binding and have therefore been omitted for clarity. Where binding to controls was detected, affinities were several orders of magnitude higher than those for the PSs 1-3 sites. Figures are given to 3 significant figures.

Compound	K_D / nM		
	PS1(JQ707375.1)	PS1ΔMotif	PS1ΔBulge
3	224000 ± 455	421000 ± 367	312000 ± 391
10	11400 ± 169	48200 ± 258	10300 ± 525
46	10200 ± 225	35500 ± 126	9650 ± 227
63	19 ± 0.5	242 ± 3	21 ± 0.43

Supplementary Table 3: Binding affinities (nM) and associated standard error of the mean of PS binding compounds used throughout, for RNA oligonucleotides PS(JQ707375.1) and its variants, PS1ΔMotif and PS1ΔBulge. Figures are given to 3 significant figures.

Compound	Normalised Anisotropy Change	Standard Error
PS1 alone	0.069	0.007
PS1 NCP	1.000	0.033
1	0.633	0.066
2	-0.167	0.050
3	0.606	0.033
4	-0.349	0.005
5	0.716	0.029
6	-0.375	0.055
7	0.914	0.017
8	0.549	0.030
9	1.014	0.021
10	0.668	0.154
11	1.182	0.057
12	2.075	0.093
13	1.205	0.040
14	0.563	0.031
15	0.716	0.026
16	0.745	0.021
17	0.388	0.025
18	-0.141	0.027
19	0.167	0.051
20	0.838	0.025
21	0.770	0.026
22	0.311	0.009
23	0.582	0.020
24	1.032	0.012
25	0.414	0.090
26	0.507	0.060
27	0.718	0.028
28	0.121	0.048
29	0.232	0.019
30	0.497	0.018
31	0.144	0.027
32	0.665	0.014
33	1.265	0.012
34	0.623	0.016
35	0.110	0.029
36	-0.393	0.033

37	0.649	0.040
38	0.585	0.009
39	-0.544	0.005
40	1.188	0.016
41	0.994	0.029
42	0.254	0.038
43	-0.131	0.015
44	0.965	0.025
45	0.367	0.034
46	0.705	0.200
47	0.728	0.029
48	0.938	0.005
49	0.885	0.011
50	1.311	0.059
51	0.129	0.026
52	0.040	0.028
53	0.596	0.005
54	1.104	0.042
55	0.807	0.063
56	0.918	0.026
57	0.141	0.014
58	0.903	0.036
59	0.314	0.018
60	0.235	0.041
61	0.647	0.014
62	0.931	0.058
63	0.496	0.036
64	1.125	0.027
65	1.451	0.018
66	0.938	0.085

Supplementary Table 4: Data associated with plate-based assembly inhibition assay using PS1 RNA oligonucleotide. Left to right – the compound used, the normalized anisotropy change with respect to the no compound control and standard error of the mean for the triplicate measurements.

Compound	Normalised Anisotropy Change	Standard Error
gRNA	0.031	0.006
gRNA NCP	0.765	0.030
3	0.916	0.016
10	1.219	0.035
46	1.594	0.011
63	0.477	0.021

Supplementary Table 5: Data associated with plate-based assembly inhibition assay using gRNA. Left to right – the compound used, the normalized anisotropy change with respect to the no compound control and standard error of the mean for the triplicate measurements.

[1] F. Fatehi, R.J. Bingham, E.C. Dykeman, N. Patel, P.G. Stockley, & R. Twarock, An intracellular model of hepatitis b viral infection: An in silico platform for comparing therapeutic strategies, Viruses. 13 (2021).

[2] N. Patel, S. Clark, E.U. Weiß, C.P. Mata, J. Bohon, E.R. Farquhar, D.P. Maskell, N.A. Ranson, R. Twarock, & P.G. Stockley, In vitro functional analysis of gRNA sites regulating assembly of hepatitis B virus, Commun. Biol. 2021 41. 4 (2021) 1–12.
